# Supplementary material for: Towards human-centric intelligent treatment planning for radiation therapy
Source: NPJ Digit Med. 2026 Jan 10;9:155. doi: 10.1038/s41746-026-02339-5 (PMC12902089; doi:10.1038/s41746-026-02339-5)
Supplement: Supplementary file 1 — Supplementary information [file 41746_2026_2339_MOESM1_ESM.pdf]

Supplementary Materials for "Towards Human-Centric Intelligent Treatment Planning for Radiation Therapy"

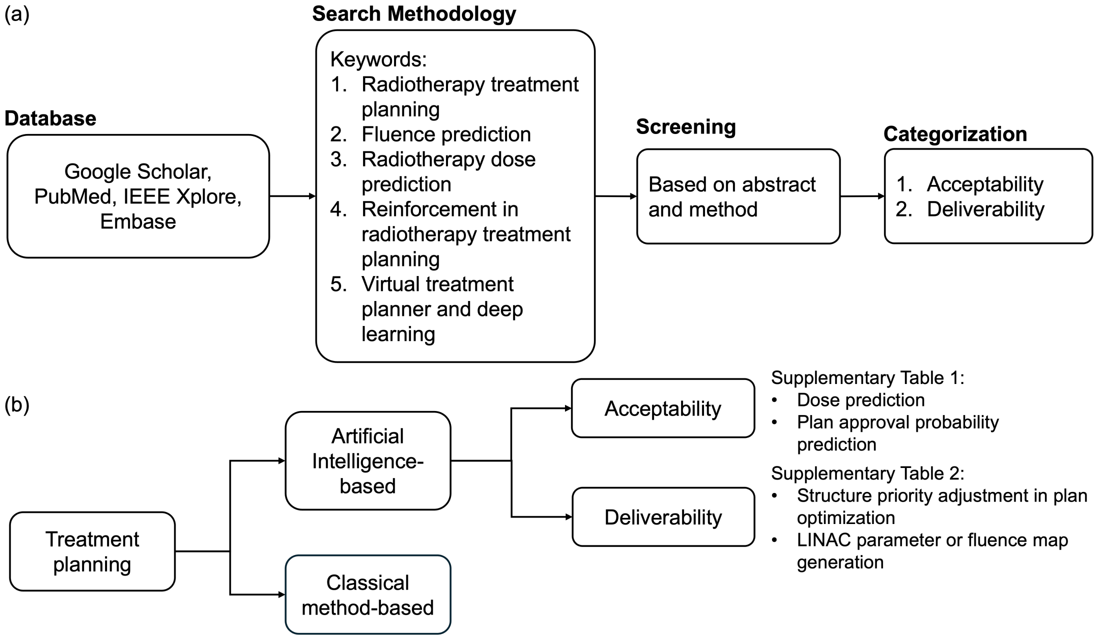

Supplementary Figure 1: Methodology of literature search. (a) Flow chart of literature search strategies and (b) Categorization of literature reviewed in this supplementary materials.

Supplementary Table 1: Automated Treatment Planning Methods Ensuring Acceptability

**Reference**	**Method**	**Dataset Size**	**Modality**	**Findings**
Nguyen et al. [1]	Used an hierarchically densely connected U-net model based on U-net and Dense-net to predict doses from inputs of ROI masks.	120 Head-and-Neck patients	VMAT	1. Predicted OARs doses were within 6.3% of the prescribed doses. 2. Prediction errors in the mean doses were within 5.1% of the prescribed doses.
Kearney et al. [2]	Used an attention-gated GAN (a deep learning framework where a generator creates data and a discriminator evaluates it) model to predict synthetic doses.	141 Prostate patients	SBRT with Cy-berknife	Attention-gated GAN achieved more realistic dose predictions compared to other state-of-art methods, with prediction errors of less than 2%.

Continued on next page

Supplementary Table 1 – continued from previous page

Reference	Method	Tumor Site	Modality	Findings
Gronberg et al. [3]	Used a U-net model to predict doses from inputs of CT volumes and ROI masks.	340 Head-and-Neck patients	IMRT	Predicted DVHs for the target and OARs showed prediction errors within 3% and 2.5 Gy, respectively, of the clinical plans.
Zimmermann et al. [4]	Used a U-net model including ResNet blocks in the decoder and encoder layers trained with another feature-based loss term to predict 3D doses given CT volumes and masks.	240 Head-and-Neck patients	IMRT	1. Prediction errors in dose and DVHs were within 1% of the clinical plans. 2. Model secured 2nd place in predicting the mean dose (inside of the external contour) and 4th place in mean absolute difference of DVH metrics compared to the baseline U-net model.
Ma et al. [5]	Used a U-net model to predict dose distribution given masks of ROIs and tandem and ovoid geometry.	45 Cervix patients	HDR BT	DVHs metrics from the predicted plans were within 8% of the ground truth plans.
Wang et al. [6]	Used a variant of U-net to predict doses from inputs of CT volumes and ROI masks.	64 Prostate patients	VMAT	Improved U-net model generated more accurate dose (with 95.8% accuracy) distributions than the standard U-net (with 95.2% accuracy).
Zhan et al. [7]	Used a multi-constraint GAN consisting of embedded U-net like generator block with dual attention module, vanilla CNN as a discriminator, and an encoder network for the perception loss to predict dose distribution given CT volumes and masks.	42 Cervical and 130 Rectal patients	IMRT	Multi-constraint GAN outperformed other state-of-art methods in nearly all PTV and OARs criteria, with prediction errors of less than 1% in DVH and dose metrics.
Gao et al. [8]	Used a U-net based GAN network to predict physician approval plans probability and plan improvement suggestion given dose distribution and ROIs masks.	68 Prostate patients	SBRT using VMAT	Proposed model achieved area under the curve of 0.97 for differentiating approved and unapproved plans as well as improved unapproved plans to achieve D98% within 3% of the ground truth.
Pastor-Serrano et al. [9]	Used a hybrid CNN-transformer model to predict dose distribution given CT volumes and projected beam geometry shape.	29 patients (Brain, Head-and-Neck, Lung, Abdomen, Pelvis, and Prostate)	VMAT	Proposed model predicted photon beams in about 50 ms and full VMAT dose distributions in about 6–12 s, with an average relative dose error of 0.75±0.36%.

Continued on next page

Supplementary Table 1 – continued from previous page

Reference	Method	Tumor Site	Modality	Findings
Yang et al. [10]	Used a TS-net model based on a vision transformer and a semantic field alignment modules to predict dose distribution given CT volumes, masks, distance image, and beam geometry shape.	120 Brain patients	IMRT	Proposed model demonstrated a mean absolute error, in dose distribution, less than 3% for PTV and less than 10% for OARs.
Jiao et al. [11]	Used a graph-convolutional network (for extracting category-specific features) and transformer-based hybrid model (for long-range sequence modeling) to predict dose distribution given only CT volumes.	120 Rectal and 42 Cervical patients	IMRT	Proposed model showed good generalizability across both rectal and cervical datasets, with prediction errors of less than 0.15 Gy in the DVH metrics.
Maes et al. [12]	Used a U-net model to predict dose distribution given CT masks followed by dose-mimicking optimization algorithm to find deliverable plans in terms of beam parameters.	60 Chest Wall patients	PBS	U-net model generated plans similar to the human-generated ones, with prediction errors of less than 2% in the PTV nodes and chest wall.
Li et al. [13]	Used a U-net model with a shared encoder to simultaneously predict dose distribution and fluence maps given CT volumes and masks.	340 NPC patients	IMRT	Predicted dose (directly generated by the network) achieved better target dose coverage and dose hot spots more than the fluence-generated dose, with a mean absolute error of less than 0.53 Gy.
Li et al. [14]	Used a cascaded U-net network with squeeze-and-excitation module to predict dose distribution given CT images, dose map, and masks of ROIs and source.	81 Cervical patients	HDR BT	Mean absolute errors in the predicted dose distributions, compared to the ground truth plans, were less than 0.35 Gy for DVHs and 0.05 Gy for dose metrics for the CTV and OARs, respectively.
Irannejad et al. [15]	Used a U-net with two input settings: one including images that contain only PTV, and the other including images that contain both PTV and OARs to predict dose distribution.	99 Glioma Tumor patients	IMRT	Using only the PTV is sufficient to predict a comparable dose distribution, with a mean absolute error of about 0.45 Gy in the DVH parameters.
Zhang et al. [16]	Used a distance-aware diffusion model to predict dose distribution given CT volumes and distance map denoting the distance from every voxel to the contour of the ROI.	119 Breast and 139 Nasopharyngeal patients	IMRT	Mean absolute errors in the predicted dose distributions, compared to the ground truth plans, were less than 2 Gy in both datasets.

Continued on next page

Supplementary Table 1 – continued from previous page

Reference	Method	Tumor Site	Modality	Findings
Yu et al. [17]	Used a U-net model with a mask guided diffusion module (to fuse CT volumes and masks effectively) to predict dose distribution given CT images and masks.	224 Cervical cases	HDR BT	Mean differences in the predicted dose parameters, compared to the ground truth plans, were about 1 Gy.
Gautam et al. [18]	Used a U-net model with attention gates in the upsampling layers to predict dose distribution given masks of ROIs.	77 Cervical patients	HDR BT	1. Mean absolute differences in the CTV and OARs dose distributions were less than 0.6 Gy. 2. Model needed less than 5 seconds to predict 3D dose distribution for a new patient.
Gao et al. [19]	Used a U-net based GAN network to predict physician aprroval probability for new plans and planning doses given geometric features and dosimetric parameters.	63 Cervical patients	HDR BT	Proposed model achieved an accuracy of about 0.7 in differentiating approved and unapproved plans, and predicted equivalent doses of 2 Gy for OARs and D90% for CTV within approximately 11% of the ground truth.
Liang et al. [20]	Used a 3D U-Net model to learn the physics principle of dose calculation to predict 3D dose distribution from inputs including projected fluence map, CT images, the radiological depth and the source-to-voxel distance.	51 Head-and-Neck patients	VMAT	Average gamma pass rates were 96.56%, 98.75%, 98.03%, and 99.30% under the criteria of 2%/2mm, 2%/3mm, 3%/2mm, and 3%/3mm (dose difference tolerance and distance-to-agreement, respectively).
Duan et al. [21]	Used an asymmetric ResNeSt model to predict 3D dose distribution from inputs including ROI masks and prediction doses.	530 Esophageal patients	IMRT	Proposed model accurately predicted 3D dose distributions with $< 5\%$ error.
Yang et al. [22]	Used a U-net model to predict fluence maps in human-AI interaction framework from inputs including initial AI plan's fluence maps, projected dose distribution, and ROI masks.	522 Whole Breast patients	WBRT	Human-AI interaction based model showed significant improvement in hotspot control over the AI plan, with an average of 25.2cc volume reduction in breast V105%.
Maniscalco et al. [23]	Used a U-net based multi-task model to predict dose distribution from inputs including CT images and treatment protocol dosimetric goals.	28 Breast patients	APBI	Proposed model had a longer training time but improved prediction accuracy compared to the task-specific baseline models, with a mean absolute percent error of $1.1033\pm0.3627\%$ with respect to the clinical plans.

Continued on next page

Supplementary Table 1 – continued from previous page

Reference	Method	Tumor Site	Modality	Findings
Dong et al. [24]	Used a graph neural network combined with a large language model (LLM) to predict 3D dose distribution from inputs including CT volumes, geometric information of OARs and PTV, and doctors' prescriptions/instructions.	40 Lung patients	IMRT	Proposed method outperformed other state-of-the-art methods, with mean percentage errors of less than 4% in the DVH metrics compared to the clinical plans.

*Abbreviations: IMRT (Intensity Modulated Radiation Therapy), VMAT (Volumetric Modulated Arc Therapy), OAR (Organ at Risk), DVH (Dose-Volume Histogram), PTV (Planning Target Volume), CTV (Clinical Target Volume), HDR (High Dose Rate), SBRT (Stereotactic Body Radiation Therapy), CT (Computed Tomography), GAN (Generative Adversarial Network), CNN (Convolutional Neural Network), PBS (Pencil Beam Scanning), U-net (U-Net Neural Network), BT (Brachytherapy), NPC (Nasopharyngeal Carcinoma), BEV (Beam's Eye View), ResNet (Residual Networks), DenseNet (Densely Connected Convolutional Networks), AUC (Area Under the Curve), 3D (Three-Dimensional), LLM (Large Language Model), APBI (Accelerated Partial Beam Irradiation), and GNN (Graph Neural Network).

Supplementary Table 2: Automated Treatment Planning Methods Ensuring Deliverability

Reference	Method	Dataset Size	Modality	Findings
Shen et al. [25]	Used a DQN model guided by rules from humans planner that takes DVHs as input and outputs organ weighting factors for the TPS.	10 Cervical patients	HDR BT	Plan quality score of DRL-generated plans improved by approximately 10% compared to clinical plans.

Continued on next page

Supplementary Table 2 – continued from previous page

**Reference**	**Method**	**Dataset Size**	**Modality**	**Findings**
Lee et al. [26]	Used a tight frame U-net to predict fluence map for each beam given given CT masks and dose distribution of each beam in the BEV.	240 Prostate patients	IMRT	Quality of synthetic plans was comparable to clinical plans, with a mean absolute error of 0.00095 in predicted fluence maps compared to clinical fluence maps.
Shen et al. [27]	Used a DQN model that takes DVHs as input and outputs organ weighting factors for the inverse optimization solver	74 Prostate patients	IMRT	Plan quality scores improved by 3.47 points compared to the initial scores from unity weighted factors.
Hrinivich and Lee [28]	Used a DQN model that takes 2D dose grid and current machine parameter values (leaf positions and dose rate) as input and outputs new machine parameters (specific leaf pair position adjustments and dose rate) to directly control LINAC.	10 Prostate patients	VMAT	Plan quality obtained from DQN VMAT plans led to slightly less coverage compared to clinical VMAT plans (83.1 vs 84.4 Gy).
Li et al. [29]	Used a Dense-Res (a combination of DenseNet and ResNet) to predict fluence maps from inputs of stacked 2D projections at 9 template beam angles.	120 Prostate patients	IMRT	Dense-Res achieved similar target coverage but had a significantly higher D0.1cc of rectum by 0.7 Gy compared to the clinical plans.
Wang et al. [30]	Used a vanilla CNN to predict field-doses distributions which were then used in customized U-net to predict fluence maps at 9 beam angles.	100 Pancreas patients	SBRT using IMRT	Model-predicted plans closely matched benchmark plans, with target dose differences of 0.1% in the mean dose, 2.1% in D95%, and organ-at-risk dose differences of 0.2% in the mean dose and 4.4% in the maximum dose.
Li et al. [31]	Used a conditional GAN to predict fluence maps from inputs of CT volumes and structures.	231 oropharyngeal patients	IMRT	After PTV coverage normalization, mean absolute differences in OAR doses were less than 0.8 Gy.
Shen et al. [32]	Used a DRL model guided by rules from humans planner that takes DVHs as input and outputs organ weighting factors for the inverse optimization solver.	74 Prostate patients	IMRT	Human-rules guided DRL method trained with eight epochs was able to perform similar as the DRL alone method trained with 100 epochs.

Continued on next page

Supplementary Table 2 – continued from previous page

**Reference**	**Method**	**Dataset Size**	**Modality**	**Findings**
Li et al. [33]	Used a conditional GAN based on ResNet blocks with three types of input features and associated models: 1. uses all 2D projections from CT for all critical structures in the BEV to predict all fluence maps, 2. uses a local small patch for each structures from a 2D projection in BEV of each beamlet to predict radiation intensity at the center of each patch, and 3. uses all small patches for each structures from 2D projections in BEV of all beamlets to predict fluence intensity.	231 Head-and-Neck patients	IMRT	1. All models showed higher ($p < 0.001$) maximum dose, conformity index, and heterogeneity index than clinical plans. 2. Local anatomical information is crucial for clinically acceptable OAR sparing, while beam angle-specific features are essential for optimal PTV coverage.
Pu et al. [34]	Used a dueling double DQN that takes dwell time of each dwell position as input and outputs which dwell time to adjust and how to adjust.	20 Cervical patients	HDR BT	Proposed method achieved comparable CTV coverage to inverse planning with simulated annealing with an absolute D90% difference of 0.24 Gy, while reducing D2cc of OARs by up to 0.23 Gy.
Sprouts et al. [35]	Used a DQN model that takes DVHs as input and outputs organ weighting factors for the inverse optimization solver.	64 Prostate patients	IMRT	DRL-based planner improved ProKnow scores by 0.4 and 0.7 points, respectively, for the two Eclipse-optimized test cases.
Wang et al. [36]	Used a double DQN model that takes DVHs as input and outputs rings structure weighting factors for the inverse optimization solver. Also used a dose prediction module based on a U-net model to set initial objectives in the inverse optimization solver to reduce the exploration time for the DQN.	114 Non-Small-Cell Lung patients	IMRT	Double DQN-generated plans were comparable to the clinical plans, with mean absolute differences in Dmax of ROIs being less than 0.95 Gy, except for the chest wall, where the clinical plans had 5.48 Gy higher Dmax.
Gao et al. [37]	Used a hierarchical DQN model that takes plan quality scores as input and outputs organ weighting factors for the inverse optimization solver.	20 Head-and-Neck patients	VMAT	Proposed method scored 125.33±11.12 on average, outperforming clinical plans (117.76±13.56).

Continued on next page

Supplementary Table 2 – continued from previous page

Reference	Method	Dataset Size	Modality	Findings
Liu et al. [38]	Used GPT-4Vision model that takes DVHs and 3D dose distributions as input and outputs organs weighting factors and objective doses for the inverse optimization solver.	17 Prostate and 13 Head-and-Neck patients	VMAT	GPT-4Vision either outperformed or matched the clinical plans, demonstrating superior target coverage and reducing organ-at-risk doses by 5 Gy on average (15% for prostate and 10-15% for head-and-neck).
Hrinivich et al. [39]	Used a DDPG model that takes cumulative dose grid at current control point, current machine parameter values (leaf positions and dose rate), and ROI masks as input and outputs new machine parameters (simultaneously all leaf pair position adjustments and dose rate) to directly control LINAC.	151 Prostate patients	VMAT	Proposed method had a similar maximum dose (63.2±0.6 Gy vs. 63.9±1.5 Gy, p=0.061) and a lower rectum dose (17.4±7.4 Gy vs. 21.0±6.0 Gy, p=0.024) compared to clinical plans.
Stephens et al. [40]	Used a Q-learning model that takes dosimetric summary of ROIs as input and outputs an increase, decrease, or no change in dose and volume objectives.	60 Head-and-Neck patients	IMRT	Proposed model reduced parotid gland dose by 7 Gy, produced plans comparable to human experts, and reduced planning time to 13.58 minutes on average.
Abrar et al. [41]	Used an actor-critic with experience replay (ACER) method that takes DVHs as input and outputs organ weighting factors for the inverse optimization solver.	83 Prostate patients	IMRT	ACER-based DRL method achieved a mean plan quality score of 8.93±0.27 across diverse patient datasets, with 93.09% of cases scoring a perfect 9.
Nusrat et al. [42]	Used large language models (8B and 70B) based on Llama family with or without retreival augment generation (RAG) and RL that takes dose-volume metrics as input and outputs organ weighting factors for the inverse optimization solver.	18 Prostate patients	IMRT	70B model demonstrated significantly improved performance, achieving approximately 16.4% (±4.5%) higher final scores than the 8B model. RAG approach outperformed the No-RAG baseline by 19.8% (±2.2%), and incorporating RL accelerated convergence.

*Abbreviations: DRL (Deep Reinforcement Learning), DQN (Deep Q-Network), DVH (Dose-Volume Histogram), TPS (Treatment Planning System), IMRT (Intensity Modulated Radiation Therapy), SBRT (Stereotactic Body Radiation Therapy), VMAT (Volumetric Modulated Arc Therapy), OAR (Organ at Risk), 3D (Three-Dimensional), DDPG (Deep Deterministic Policy Gradient), ROI (Region of Interest), and LINAC (Linear Accelerator), and GPT (Generative Pre-trained Transformer).

References

[1] Dan Nguyen, Xun Jia, David Sher, Mu-Han Lin, Zohaib Iqbal, Hui Liu, and Steve Jiang. 3d radiotherapy dose prediction on head and neck cancer patients with a hierarchically densely connected u-net deep learning architecture. *Physics in Medicine & Biology*, 64(6):065020, 2019. doi: 10.1088/1361-6560/ab039b.

[2] Vasant Kearney, Jason W. Chan, Tianqi Wang, Alan Perry, Martina Descovich, Olivier Morin, Sue S. Yom, and Timothy D. Solberg. Dosegan: a generative adversarial network for synthetic dose prediction using attention-gated discrimination and generation. *Scientific Reports*, 10(1):11073, 2020. doi: 10.1038/s41598-020-68062-7.

[3] Mary P. Gronberg, Skylar S. Gay, Tucker J. Netherton, Dong Joo Rhee, Laurence E. Court, and Carlos E. Cardenas. Dose prediction for head and neck radiotherapy using a three-dimensional dense dilated u-net architecture. *Medical physics*, 48(9):5567–5573, 2021. doi: 10.1002/mp.14827.

[4] Lukas Zimmermann, Erik Faustmann, Christian Ramsl, Dietmar Georg, and Gerd Heilemann. Dose prediction for radiation therapy using feature-based losses and one cycle learning. *Medical Physics*, 48(9):5562–5566, 2021. doi: 10.1002/mp.14774.

[5] Ming Ma, Elizabeth Kidd, Benjamin P. Fahimian, Bin Han, Thomas R. Niedermayr, Dimitre Hristov, Lei Xing, and Yong Yang. Dose prediction for cervical cancer brachytherapy using 3-d deep convolutional neural network. *IEEE Transactions on Radiation and Plasma Medical Sciences*, 6(2):214–221, 2021. doi: 10.1109/TRPMS.2021.3057982.

[6] Jianyong Wang, Junjie Hu, Ying Song, Qiang Wang, Xiaozhi Zhang, Sen Bai, and Zhang Yi. Vmat dose prediction in radiotherapy by using progressive refinement unet. *Neurocomputing*, 488:528–539, 2022. doi: 10.1016/j.neucom.2021.11.061.

[7] Bo Zhan, Jianghong Xiao, Chongyang Cao, Xingchen Peng, Chen Zu, Jiliu Zhou, and Yan Wang. Multi-constraint generative adversarial network for dose prediction in radiotherapy. *Medical Image Analysis*, 77:102339, 2022. doi: 10.1016/j.media.2021.102339.

[8] Yin Gao, Chenyang Shen, Yesenia Gonzalez, and Xun Jia. Modeling physician's preference in treatment plan approval of stereotactic body radiation therapy of prostate cancer. *Physics in Medicine & Biology*, 67(11):115012, 2022. doi: 10.1088/1361-6560/ac6f6c.

[9] Oscar Pastor-Serrano, Peng Dong, Charles Huang, Lei Xing, and Zoltán Perkó. Sub-second photon dose prediction via transformer neural networks. *Medical Physics*, 50(5):3159–3171, 2023. doi: 10.1002/mp.16379.

[10] Jinna Yang, Yuqian Zhao, Fan Zhang, Miao Liao, and Xiaoyu Yang. Deep learning architecture with transformer and semantic field alignment for voxel-level dose prediction on brain tumors. *Medical Physics*, 50(2):1149–1161, 2023. doi: 10.1002/mp.16180.

[11] Zhengyang Jiao, Xingchen Peng, Yan Wang, Jianghong Xiao, Dong Nie, Xi Wu, Xin Wang, Jiliu Zhou, and Dinggang Shen. Transdose: Transformer-based radiotherapy dose prediction from ct images guided by super-pixel-level gcn classification. *Medical Image Analysis*, 89:102902, 2023. doi: 10.1016/j.media.2023.102902.

[12] Dominic Maes, Mats Holmstrom, Rasmus Helander, Jatinder Saini, Christine Fang, and Stephen R. Bowen. Automated treatment planning for proton pencil beam scanning using deep learning dose prediction and dose-mimicking optimization. *Journal of Applied Clinical Medical Physics*, 24(10):e14065, 2023. doi: 10.1002/acm2.14065.

[13] Yongbao Li, Wenwen Cai, Fan Xiao, Xuanru Zhou, Jiajun Cai, Linghong Zhou, Wen Dou, and Ting Song. Simultaneous dose distribution and fluence prediction for nasopharyngeal carcinoma imrt. *Radiation Oncology*, 18(1):110, 2023. doi: 10.1186/s13014-023-02271-w.

[14] Zhen Li, Zhenyu Yang, Jiayu Lu, Qingyuan Zhu, Yanxiao Wang, Mengli Zhao, Zhaobin Li, and Jie Fu. Deep learning-based dose map prediction for high-dose-rate brachytherapy. *Physics in Medicine & Biology*, 68(17):175015, 2023. doi: 10.1088/1361-6560/accf2d.

[15] Maziar Irannejad, Iraj Abedi, Vida Darbaghi Lonbani, and Maryam Hassanvand.

Deep-neural network approaches for predicting 3d dose distribution in intensity-modulated radiotherapy of the brain tumors. *Journal of Applied Clinical Medical Physics*, 25(3):e14197, 2024. doi: 10.1002/acm2.14197.

[16] Yiwen Zhang, Chuanpu Li, Liming Zhong, Zeli Chen, Wei Yang, and Xuetao Wang. Dosediff: distance-aware diffusion model for dose prediction in radiotherapy. *IEEE Transactions on Medical Imaging*, 2024. doi: 10.1109/TMI.2024.3383423.

[17] Lang Yu, Wenjun Zhang, Jie Zhang, Qi Chen, Lu Bai, Nan Liu, Tingtian Pang, Bo Yang, and Jie Qiu. A cnn-based dose prediction method for brachytherapy treatment planning of patients with cervical cancer. *Journal of Radiation Research and Applied Sciences*, 17(3):101013, 2024. doi: 10.1016/j.jrras.2024.101013.

[18] Suman Gautam, Alexander FI Osman, Dylan Richeson, Somayeh Gholami, Binod Manandhar, Sharmin Alam, and William Y. Song. Attention 3d unet for dose distribution prediction of high-dose-rate brachytherapy of cervical cancer: Intracavitary applicators. *Journal of Applied Clinical Medical Physics*, page e14568, 2024. doi: 10.1002/acm2.14568.

[19] Yin Gao, Yesenia Gonzalez, Chika Nwachukwu, Kevin Albuquerque, and Xun Jia. Predicting treatment plan approval probability for high-dose-rate brachytherapy of cervical cancer using adversarial deep learning. *Physics in Medicine & Biology*, 69(9):095010, 2024. doi: 10.1088/1361-6560/abf1e3.

[20] Bin Liang, Wenlong Xia, Ran Wei, Yuan Xu, Zhiqiang Liu, and Jianrong Dai. A deep learning-based dose calculation method for volumetric modulated arc therapy. *Radiation Oncology*, 19(1):141, 2024. doi: 10.1186/s13014-024-02534-2.

[21] Yanhua Duan, Jiyong Wang, Puyu Wu, Yan Shao, Hua Chen, Hao Wang, Hongbin Cao, et al. As-nest: A novel 3d deep learning model for radiation therapy dose distribution prediction in esophageal cancer treatment with multiple prescriptions. *International Journal of Radiation Oncology* Biology* Physics*, 119(3):978–989, 2024. doi: 10.1016/j.ijrobp.2023.12.001.

[22] Dongrong Yang, Cameron Murr, Xinyi Li, Sua Yoo, Rachel Blitzblau, Susan

McDuff, Sarah Stephens, Q. Jackie Wu, Qiuwen Wu, and Yang Sheng. Understanding and modeling human-ai interaction of artificial intelligence tool in radiation oncology clinic using deep neural network: a feasibility study using three year prospective data. *Physics in Medicine & Biology*, 69(22):225018, 2024. doi: 10.1088/1361-6560/ad8e29.

[23] Austen Maniscalco, Ezek Mathew, David Parsons, Justin Visak, Mona Arbab, Prasanna Alluri, Xingzhe Li, and et al. Multimodal radiotherapy dose prediction using a multi-task deep learning model. *Medical Physics*, 51(6):3932–3949, 2024. doi: 10.1002/mp.17115.

[24] Zehao Dong, Yixin Chen, Hiram Gay, Yao Hao, Geoffrey D. Hugo, Pamela Samson, and Tianyu Zhao. Large-language-model empowered 3d dose prediction for intensity-modulated radiotherapy. *Medical Physics*, 2024. doi: 10.1002/mp.17416.

[25] Chenyang Shen, Yesenia Gonzalez, Peter Klages, Nan Qin, Hyunuk Jung, Liyuan Chen, Dan Nguyen, Steve B. Jiang, and Xun Jia. Intelligent inverse treatment planning via deep reinforcement learning, a proof-of-principle study in high dose-rate brachytherapy for cervical cancer. *Physics in Medicine Biology*, 64 (11):115013, 2019. doi: 10.1088/1361-6560/ab1c71.

[26] Hoyeon Lee, Hojin Kim, Jungwon Kwak, Young Seok Kim, Sang Wook Lee, Seungryong Cho, and Byungchul Cho. Fluence-map generation for prostate intensity-modulated radiotherapy planning using a deep-neural-network. *Scientific Reports*, 9(1):15671, 2019. doi: 10.1038/s41598-019-52159-7.

[27] Chenyang Shen, Dan Nguyen, Liyuan Chen, Yesenia Gonzalez, Rafe McBeth, Nan Qin, Steve B. Jiang, and Xun Jia. Operating a treatment planning system using a deep-reinforcement learning-based virtual treatment planner for prostate cancer intensity-modulated radiation therapy treatment planning. *Medical Physics*, 47 (6):2329–2336, 2020. doi: 10.1002/mp.14135.

[28] William Thomas Hrinivich and Junghoon Lee. Artificial intelligence-based radiotherapy machine parameter optimization using reinforcement learning. *Medical Physics*, 47(12):6140–6150, 2020. doi: 10.1002/mp.14149.

[29] Xinyi Li, Jiahan Zhang, Yang Sheng, Yushi Chang, Fang-Fang Yin, Yaorong Ge, Q. Jackie Wu, and Chunhao Wang. Automatic imrt planning via static field fluence prediction (aip-sffp): a deep learning algorithm for real-time prostate treatment planning. *Physics in Medicine & Biology*, 65(17):175014, 2020. doi: 10.1088/1361-6560/aba5eb.

[30] Wentao Wang, Yang Sheng, Chunhao Wang, Jiahan Zhang, Xinyi Li, Manisha Palta, Brian Czito, and et al. Fluence map prediction using deep learning models–direct plan generation for pancreas stereotactic body radiation therapy. *Frontiers in Artificial Intelligence*, 3:68, 2020. doi: 10.3389/frai.2020.00068.

[31] Xinyi Li, Chunhao Wang, Yang Sheng, Jiahan Zhang, Wentao Wang, Fang-Fang Yin, Qiuwen Wu, Q. Jackie Wu, and Yaorong Ge. An artificial intelligence-driven agent for real-time head-and-neck imrt plan generation using conditional generative adversarial network (cgan). *Medical Physics*, 48(6):2714–2723, 2021. doi: 10.1002/mp.14770.

[32] Chenyang Shen, Liyuan Chen, Yesenia Gonzalez, and Xun Jia. Improving efficiency of training a virtual treatment planner network via knowledge-guided deep reinforcement learning for intelligent automatic treatment planning of radiotherapy. *Medical Physics*, 48(4):1909–1920, 2021. doi: 10.1002/mp.14745.

[33] Xinyi Li, Yaorong Ge, Qiuwen Wu, Chunhao Wang, Yang Sheng, Wentao Wang, Hunter Stephens, Fang-Fang Yin, and Q. Jackie Wu. Input feature design and its impact on the performance of deep learning models for predicting fluence maps in intensity-modulated radiation therapy. *Physics in Medicine & Biology*, 67(21):215009, 2022. doi: 10.1088/1361-6560/ac9882.

[34] Gang Pu, Shan Jiang, Zhiyong Yang, Yuanjing Hu, and Ziqi Liu. Deep reinforcement learning for treatment planning in high-dose-rate cervical brachytherapy. *Physica Medica*, 94:1–7, 2022. doi: 10.1016/j.ejmp.2021.12.009.

[35] Damon Sprouts, Yin Gao, Chao Wang, Xun Jia, Chenyang Shen, and Yujie Chi. The development of a deep reinforcement learning network for dose-volume-constrained treatment planning in prostate cancer intensity modulated

radiotherapy. *Biomedical Physics & Engineering Express*, 8(4):045008, 2022. doi: 10.1088/2057-1976/ac7c65.

[36] Hanlin Wang, Xue Bai, Yajuan Wang, Yanfei Lu, and Binbing Wang. An integrated solution of deep reinforcement learning for automatic imrt treatment planning in non-small-cell lung cancer. *Frontiers in Oncology*, 13:1124458, 2023. doi: 10.3389/fonc.2023.1124458.

[37] Yin Gao, Yang Kyun Park, and Xun Jia. Human-like intelligent automatic treatment planning of head and neck cancer radiation therapy. *Physics in Medicine & Biology*, 69(11):115049, 2024. doi: 10.1088/1361-6560/abddff.

[38] Sheng Liu, Oscar Pastor-Serrano, Yizheng Chen, Matthew Gopaulchan, Weixing Liang, Mark Buyyounouski, Erqi Pollom, et al. Automated radiotherapy treatment planning guided by gpt-4vision. *arXiv preprint arXiv:2406.15609*, 2024.

[39] William T. Hrinivich, Mahasweta Bhattacharya, Lina Mekki, Todd McNutt, Xun Jia, Heng Li, Daniel Y. Song, and Junghoon Lee. Clinical vmat machine parameter optimization for localized prostate cancer using deep reinforcement learning. *Medical Physics*, 51(6):3972–3984, 2024. doi: 10.1002/mp.16097.

[40] Hunter Stephens, Xinyi Li, Yang Sheng, Qiuwen Wu, Yaorong Ge, and Q. Jackie Wu. A reinforcement learning agent for head and neck intensity-modulated radiation therapy. *Frontiers in Physics*, 12:1331849, 2024. doi: 10.3389/fphy.2024.1331849.

[41] Md Mainul Abrar, Parvat Sapkota, Damon Sprouts, Xun Jia, and Yujie Chi. Actor critic with experience replay-based automatic treatment planning for prostate cancer intensity modulated radiotherapy. *arXiv preprint arXiv:2502.00346*, 2025.

[42] H. Nusrat, B. Luo, R. Hall, J. Kim, H. Bagher-Ebadian, A. Doemer, B. Movsas, and K. Thind. Autonomous radiotherapy treatment planning using dola: A privacy-preserving, llm-based optimization agent. *arXiv preprint arXiv:2503.17553*, 2025.
